# Enhanced Therapeutic Potential of Hybrid Exosomes Loaded with Paclitaxel for Cancer Therapy

**DOI:** 10.3390/ijms25073645

**Published:** 2024-03-25

**Authors:** Xuan Wang, Dongdong Li, Gaotian Li, Jinda Chen, Yi Yang, Lijun Bian, Jingying Zhou, Yongge Wu, Yan Chen

**Affiliations:** 1 National Engineering Laboratory for AIDS Vaccine, School of Life Sciences, Jilin University, Changchun 130012, China; xuanwang18@mails.jlu.edu.cn (X.W.); ldd19@mails.jlu.edu.cn (D.L.); ligt22@mails.jlu.edu.cn (G.L.); chenjd23@mails.jlu.edu.cn (J.C.); yiy23@mails.jlu.edu.cn (Y.Y.); bianlj21@mails.jlu.edu.cn (L.B.); jyzhou20@mails.jlu.edu.cn (J.Z.); ygwu@jlu.edu.cn (Y.W.); 2 Key Laboratory for Molecular Enzymology and Engineering of Ministry of Education, School of Life Sciences, Jilin University, Changchun 130012, China

**Keywords:** MSC-derived exosomes, hybrid system, colorectal cancer, tumor microenvironment, T cells

## Abstract

The advancement of exosome studies has positioned engineered exosomes as crucial biomaterials for the development of advanced drug delivery systems. This study focuses on developing a hybrid exosome system by fusing mesenchymal stem cells (MSCs) exosomes with folate-targeted liposomes. The aim was to improve the drug loading capacity and target modification of exosome nanocarriers for delivering the first-line chemotherapy drug paclitaxel (PTX) and its effectiveness was assessed through cellular uptake studies to evaluate its ability to deliver drugs to tumor cells in vitro. Additionally, in vivo experiments were conducted using a CT26 tumor-bearing mouse model to assess the therapeutic efficacy of hybrid exosomes loaded with PTX (ELP). Cellular uptake studies demonstrated that ELP exhibited superior drug delivery capabilities to tumor cells in vitro. Moreover, in vivo experiments revealed that ELP significantly suppressed tumor growth in the CT26 tumor-bearing mouse model. Notably, for the first time, we examined the tumor microenvironment following intratumoral administration of ELP. We observed that ELP treatment activated CD4^+^ and CD8^+^ T cells, reduced the expression of M2 type tumor-associated macrophages (TAMs), polarized TAMs towards the M1 type, and decreased regulatory T cells (Tregs). Our research highlights the considerable therapeutic efficacy of ELP and its promising potential for future application in cancer therapy. The development of hybrid exosomes presents an innovative approach to enhance drug delivery and modulate the tumor microenvironment, offering exciting prospects for effective cancer treatment strategies.

## 1. Introduction

The quest for more safe and efficient drug delivery systems (DDS) is spurring the exploration of bioinspired approaches to surmount barriers hindering DDS access to target tissues. There is a growing interest in customized vectors derived from components of human tissues and cells, such as cell membranes derived from the patient’s own cells. This emerging field aims to create biocamouflaged vectors with extended circulation time, enhanced ability to traverse biological barriers, reduced immunogenicity, and intrinsic targeting capabilities [1,2]. However, current methodologies for engineering cell-derived products into versatile DDS remain limited, presenting a significant challenge. In contrast, synthetic nanocarriers like liposomes have long surpassed biogenic DDS in terms of versatility, production yield, drug loading efficiency, surface modification, and standardization [3]. The fusion of liposomes’ ease of production and modification with the intrinsic functionalities of biogenic delivery systems—biocompatibility, targeting, and uptake properties—holds immense potential. This convergence would yield bioengineered DDS that combine the strengths of both synthetic and biological entities, promising enhanced therapeutic outcomes.

Exosomes, first coined by Johnstone et al. in 1987, are released through the fusion of multivesicular endosomal bodies (MVBs) with the plasma membrane [4]. Typically ranging from 30 to 150 nm in diameter, exosomes exhibit a bilayer membrane structure akin to liposomes, resembling miniature versions of their parent cells in function and characteristics [5]. Their natural sourcing, low immunogenicity, and ability to target homotypic sources, including passage through the blood–brain barrier (BBB), have sparked interest in their potential as drug delivery agents for therapeutic targets across diverse diseases [6]. Research has demonstrated the promise of exosomes derived from various cell lines in drug delivery for different conditions. For instance, exosomes derived from the mouse macrophage cell line RAW264.7 have been investigated for treating Parkinson’s disease and pulmonary metastases [7]. Similarly, exosomes encapsulating curcumin derived from the mouse lymphoma cell line EL-4 have shown enhanced anti-inflammatory effects [8]. Additionally, exosomes derived from PANC-1 and U937 cells have been explored for delivering paclitaxel (PTX) to pancreatic cancer sites, while exosomes loaded with doxorubicin derived from mouse immature dendritic cells (imDCs) have been utilized for targeted delivery [9]. The clinical trials of exosomes in drug delivery systems were mostly Phase 1 studies. One study supported by Codiak BioSciences company evaluated the exosomes loaded with CDK-004 (exoASO-STAT6)’s safety, tolerability, and initial antitumor activity in patients with advanced hepatocellular carcinoma (HCC) and patients with liver metastases from either primary gastric cancer or colorectal cancer (NCT05375604). Additionally, MSC-derived exosomes were used to deliver AGLE-102 for burn wounds (NCT05078385). However, despite their potential benefits, there are concerns regarding tumor-derived exosomes promoting tumor growth and metastasis, posing safety risks. Therefore, identifying safe maternal source exosomes for tumor treatment remains a crucial exploration direction.

Mesenchymal stem cells (MSCs) have emerged as pivotal regulators of innate and adaptive immunity, contributing to immune homeostasis in various diseases [10]. Preclinical studies have shown that MSCs can suppress tumor growth and metastasis by suppressing tumor angiogenesis through the release of antiangiogenic factors in models of lung metastasis, melanoma, colon cancer, prostate cancer, and ovarian cancer, and MSC also mediated downregulation of the PDGF/PDGFR axis, suppressing glioma angiogenesis [11,12]. Nonetheless, their relatively large size raises challenges, such as potential entrapment in the lungs, hindering their clinical use. Despite this, MSCs are prolific producers of exosomes, making them an attractive option for drug delivery. Furthermore, studies have demonstrated that MSC-derived Exosomes (Exo) play an important role in regulating the function of multiple immune-related cell types, such as natural killer T (NKT) cells, regulatory T cells, and macrophages [13]. Exosomes can also encapsulate desired therapeutic cargoes, such as miRNAs, proteins, and drugs. However, traditional drug loading methods, such as electroporation, suffer from low drug loading efficiency and poor yield, limiting their clinical potential. To address these challenges, leveraging liposome engineering technologies to engineer exosomes has been proposed as a solution to overcome these limitations. Up to now, there are few reports on the hybridization of Exo with liposomes for carrying antitumor drugs.

The natural compound paclitaxel, derived from taxus, exerts its antimitotic effects by targeting tubulin and has been approved by the FDA as the first chemotherapeutic agent sourced from a plant [14]. Since its initial discovery in 1967, research and development surrounding paclitaxel has remained ceaseless. At present, the clinical experimental studies of paclitaxel are mainly focused on the combination of paclitaxel and other chemotherapy drugs to control tumor recurrence and metastasis; clinical trials such as albumin-bound paclitaxel combined with gemcitabine first-line inoperable pancreatic cancer (NCT05035147) and combined with carboplatin versus epirubicin and docetaxel for triple-negative breast cancer (NCT04136782) are in Phase 4 study. Currently, a small number of paclitaxel and its derivatives are on the market, such as TAXOTERE^®^ (1996), Abraxane^®^ (2005), and Jevtana^®^ (2010). However, TAXOTERE^®^ was a paclitaxel injection which due to paclitaxel’s poor solubility, must use polyoxyethyl castor oil to increase the solubility, which may easily cause adverse reactions. Abraxane^®^ and Jevtana^®^ were paclitaxel liposomes but also may cause an allergic reaction to paclitaxel. Moreover, due to the lack of targeting, all types of paclitaxel have a strong systemic toxic effect. Therefore, it is important to develop a safer and more efficient drug delivery system.

**Scheme 1:**
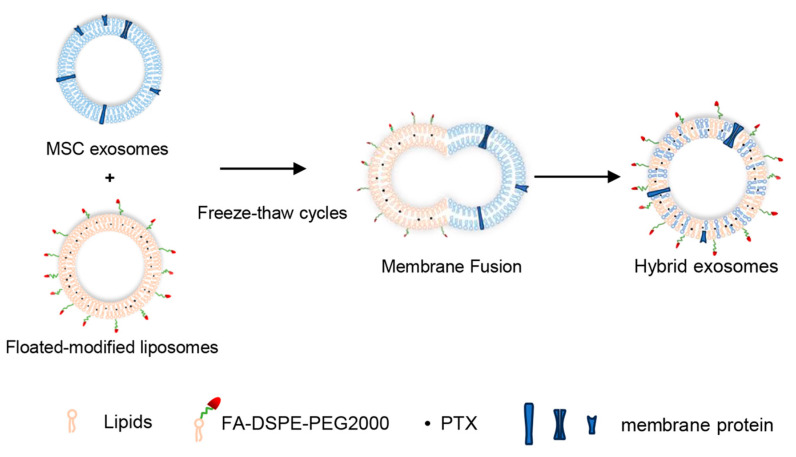
Hybrid exosome synthesis mechanisms.

Due to these previously stated concerns and considering the numerous similarities between liposomes and exosomes, in this research, we designed a tumor-target hybrid exosome vesicles, composed of Exo and folate-modified targeting liposomes, for antitumor drug PTX delivery (Scheme 1). It was expected that the hybrid vectors had better drug loading capacity, stability, and tumor targeting capacity. The antitumor effect of the hybrid vector loaded with PTX and its regulation of the tumor immune microenvironment were investigated in a mouse model.

## 2. Results

### 2.1. Physico-Chemical Properties of Hybrid Exosomes

**Figure 1:**
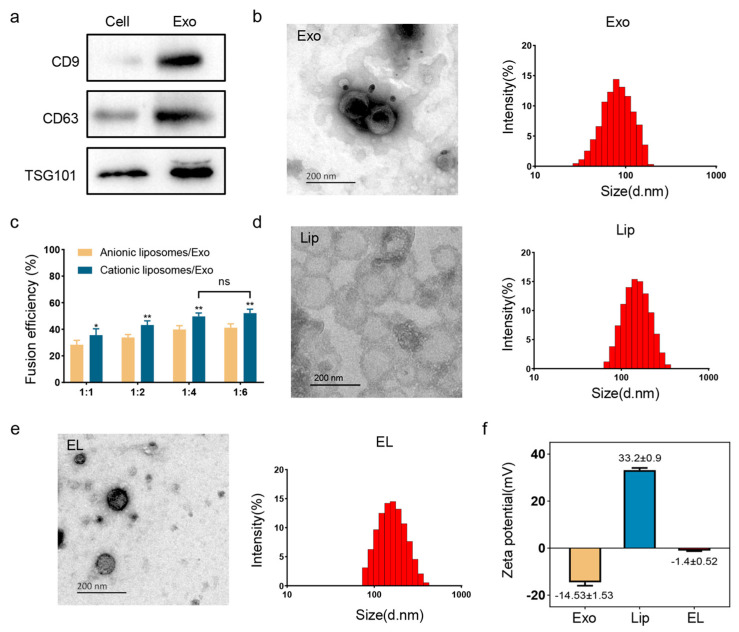
Characterization of MSC-derived exosomes (Exo), folate-modified liposomes (Lip), and hybrid exosomes (EL). (**a**) Western blot analysis of MSC cells and their exosomes for the indicated proteins. (**b**) Transmission electron microscopy morphology and size distribution of Exo. (**c**) The fusion efficiency of Exo and liposomes with different surface charges by FRET. (**d**,**e**) Transmission electron microscopy morphology and size distribution of Lip and EL. (**f**) Representative graphs of the surface zeta potential of Exo, Lip, and EL. Scale bar = 200 nm. * *p* < 0.05, ** *p* < 0.01, ns = no significance.

Exo were prepared using the ultracentrifugation method and characterized via Western blot analysis to ascertain the presence of the exosomal marker proteins CD9, TSG101, and CD63 (Figure 1a). To further characterize the identity of exosomes, Transmission Electron Microscope (TEM) and dynamic light scattering (DLS) were employed. TEM analysis revealed a cup-shaped morphology of exosomes with a discernible lipid layer (Figure 1b). DLS measurements of Exo unveiled a size of approximately 90 nm and a zeta potential of −13 mV (Figure 1b,f).

The hybrid exosomes fusing with liposomes (EL) were prepared using the freeze–thaw method. Folate-modified targeting liposomes with different surface charges (positive or negative) and Exo were fused at a volume ratio of 1:1, 1:2, 1:4, and 1:6, then the fusion efficiency was detected using FRET technology. The results showed that cationic liposomes had a better fusion ability than anionic liposomes (*p* < 0.05), and fusion efficiency reached a maximum value at 1:4 (Figure 1c). So, the hybrid exosome vesicles were prepared with folate-modified cationic liposomes (Lip) and Exo at a volume ratio of 1:4 for the following experiments. TEM analysis of Lip and EL showed a characteristic spherical shape (Figure 1d,e). The average hydrodynamic diameters of Lip and EL were 138.5 nm (with a zeta potential of 33.2 mV) and 150 nm (with a zeta potential of −1.4 mV), respectively (Figure 1d–f). As shown in Figure 1f, the change of zeta potential also indirectly demonstrated the fusion between Exo and Lip.

### 2.2. The Drug Carrying Capacity, Stability, and Safty of Hybrid Exosomes

**Figure 2:**
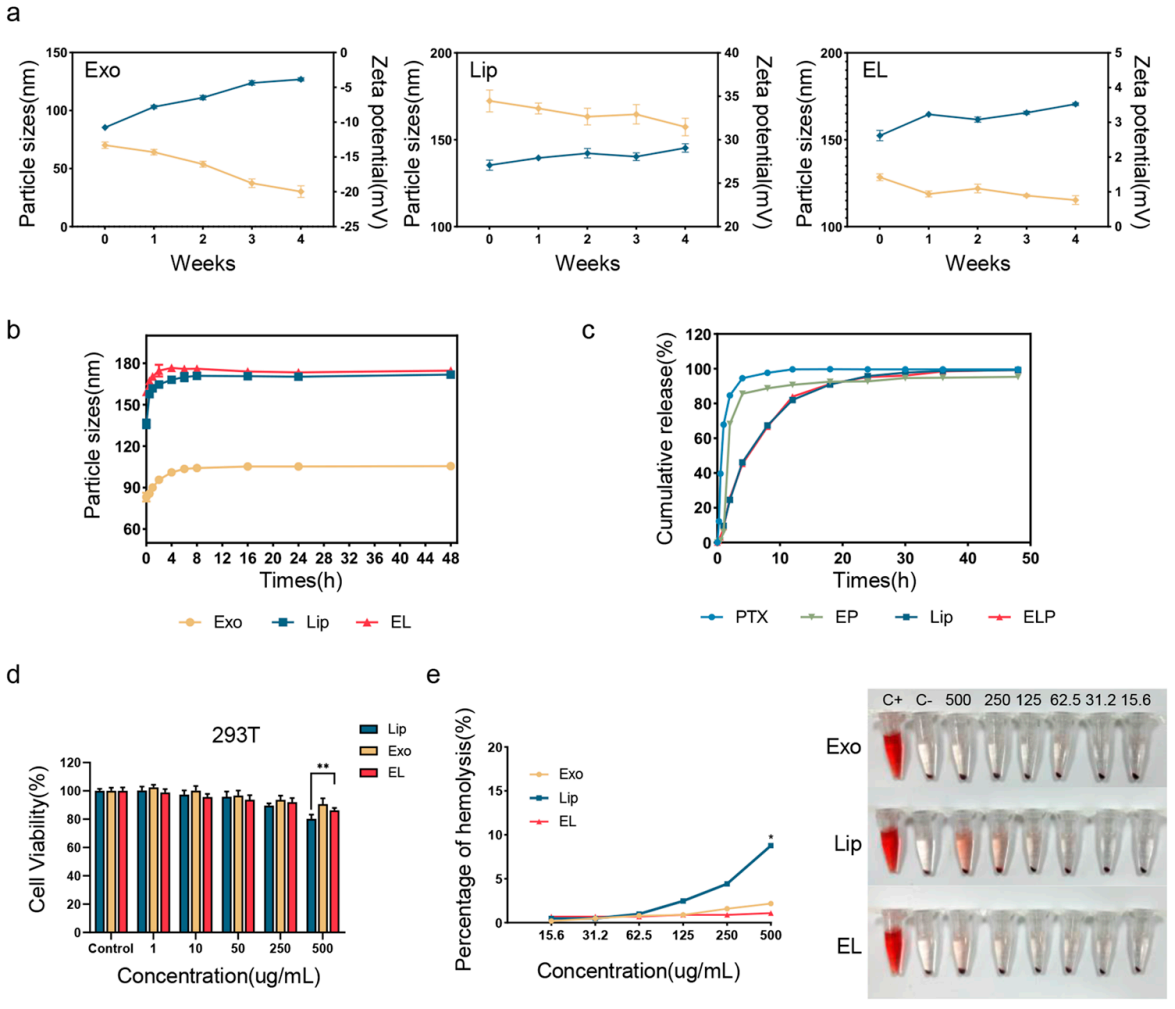
The stability, drug release, and safety of Exo, Lip, and EL. (**a**) The stability of Exo, Lip, and EL in 4 weeks at 4 °C. (**b**) The particle size of Exo, Lip, and EL in 48 h exposed to 10% FBS medium. (**c**) The release of PTX from Exo, Lip, and EL at 37 °C in 48 h. (**d**) The cytotoxicity of Lip, Exo, and EL in 293T cells. (**e**) RBC hemolysis in response to various Exo, Lip, and EL concentrations (**left**), photographs of Exo, Lip, and ELP concentrations after 2 h of incubation with RBCs (**right**). * *p* < 0.05, ** *p* < 0.01.

One of the main limiting factors for the clinical use of exosomes is their low drug-carrying capacity. The PTX loading contents (LC) in Exo and EL were determined by HPLC. The results showed that EL had a significantly higher PTX carrying capacity (2.2% LC) than Exo (1.0% LC). The particle sizes and surface zeta potential of Exo, Lip, and EL were detected during four weeks at 4 °C. The average particle size of Exo was significantly increased from 85.4 nm to 126.9 nm, while there were negligible changes in EL (Figure 2a). When exposed to 10% FBS medium, the particle sizes of EL demonstrated no obvious change (Figure 2b). These results indicated that the hybrid exosome vesicles we prepared had good stability.

The in vitro release cures of PTX from Exo, Lip, and EL under physiological conditions of 37 °C and pH 7.4 are shown in Figure 2c. The cumulative PTX release from Exo exceeded 80% within 4 h, characterized by a rapid, burst release profile, which may not favor the effective accumulation of therapeutic agents within tumor tissues. Conversely, the EL group exhibited a sustained drug release pattern over a span of 48 h.

We subsequently assessed the safety of EL in vitro. HEK293T cells were incubated with different concentrations of Lip, Exo, and EL for 24 h, and the cell viability is shown in Figure 2d. When the concentration increased to 500 μg/mL, the Lip group exhibited significant cytotoxicity, which might be attributed to its positive charge, while Exo and EL showed more than 90% cell viability at the same concentration. Correspondingly, the hemolysis assay showed similar results (Figure 2e). The Exo, Lip, and EL were incubated with erythrocytes for 2 h at various concentrations. Notably, the singular Lip group at 500 μg/mL caused significant hemolysis, but the Exo and EL did not show hemolysis. These results showed that the safety of hybrid exosome vesicles was better than that of Lip.

### 2.3. The Ability to Enhance Cellular Uptake and Apoptosis of Tumor Cells In Vitro

**Figure 3:**
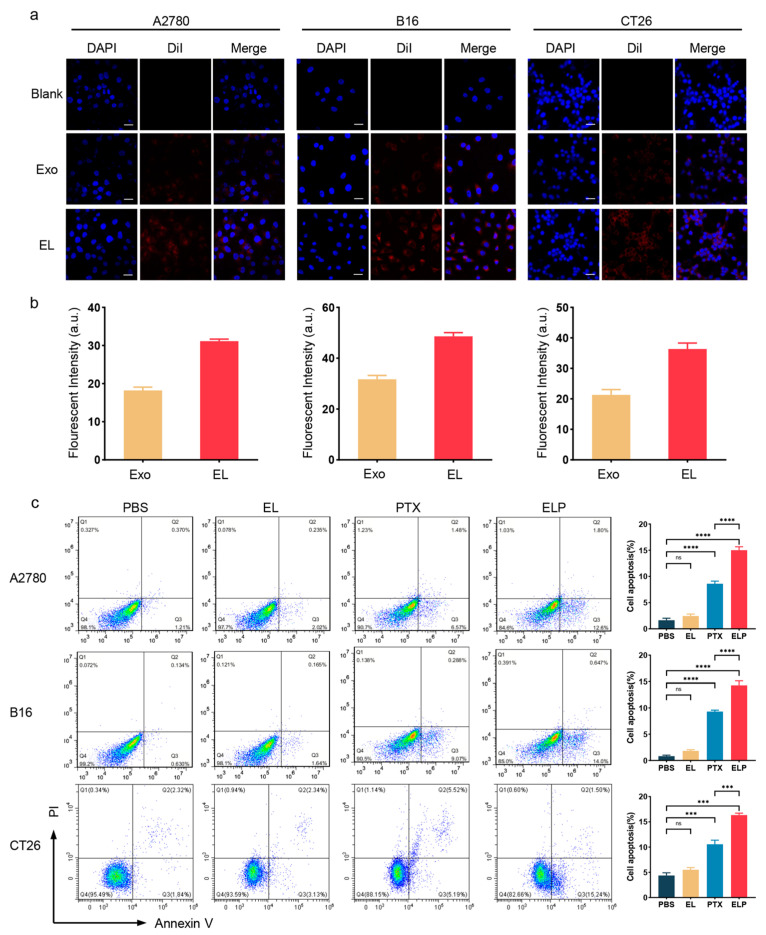
In vitro assays of the Exo, EL, and ELP. (**a**) The CLSM images of Exo and EL in A2780 cell, B16 cell, and CT26 cell. DiI, a lipophilic red fluorescence dye, was selected to label the Exo and EL. DAPI, a blue fluorescence dye, was selected to label the cell nucleus. Scale bar: 50 μm. (**b**) The quantification of CLSM by ImageJ software (Version 1.54g). (**c**) Flow cytometry of cell apoptosis after incubation with EL, PTX, and ELP in A2780, B16, and CT26 tumor cells (*n* = 3), *** *p* < 0.001, **** *p* < 0.0001, ns = no significance.

Earlier investigations have delineated the targeted affinity of MSC exosomes towards tumors [15]. To evaluate the tumor-targeting capacity of hybrid exosomes, we compared the cellular uptake efficiency of Exo and EL by tumor cells. The cellular uptake was performed using different tumor cell lines. DiI, a lipophilic dye, was selected to label the Exo and EL as previously described. Following the incubation of Exo and EL with A2780, B16, and CT26 cells for 4 h, fluorescence microscopy was employed for visualization. Cell nuclei were stained with DAPI, rendering blue fluorescence, while red fluorescence denoted the localization of Exo or EL. Findings revealed that Exo exhibited a certain degree of cellular internalization, while the amalgamation with folate-modified liposomes markedly enhanced the ability of target recognition and uptake (Figure 3a). The quantification results are presented in Figure 3b. We also tested the targeting capacity of hybrid exosomes in low folate receptor expression Human Embryonic Kidney 293 cells and found a decrease in the targeted hybrid exosomes in 293 cells, which illustrated the folate-modified’ effectivity (Figure S1).

To further evaluate whether the hybrid exosomes loaded with PTX had better antitumor activity, we incubated EL, 1 μM PTX, and EL loaded with 1 μM PTX (ELP) with A2780, B16, and CT26 cell lines for 24 h, respectively, and then collected cells for apoptosis analysis using Annexin V-PI staining (Figure 3c). The results showed that EL-carrying PTX could significantly enhance the apoptosis of tumor cells compared with free PTX. 

### 2.4. The Ability to Enhance Antitumor Efficacy In Vivo

**Figure 4:**
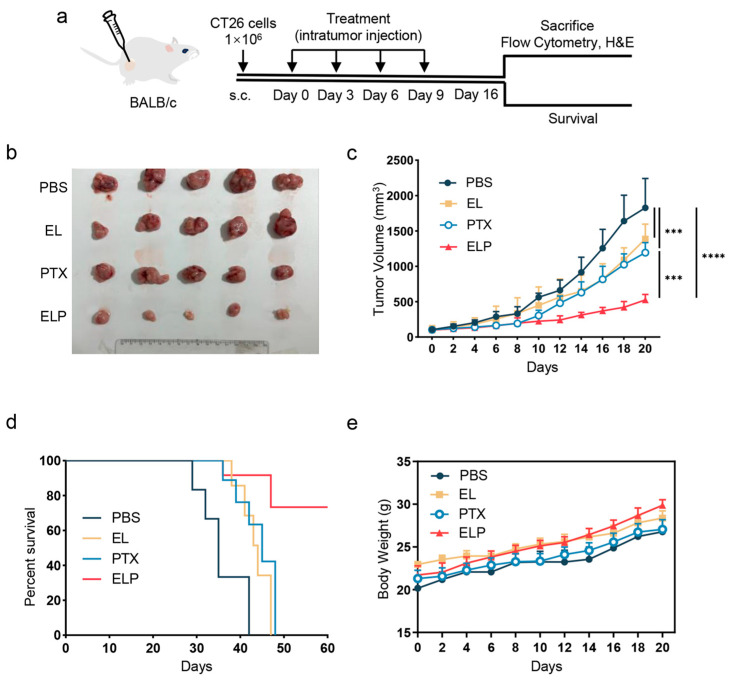
In vivo antitumor effects of ELP. (**a**) Experimental scheme diagram. (**b**) The image of excised CT26 tumors at day 16. (**c**) Tumor growth curves. (**d**) Kaplan–Meier analysis of animal endpoint survival after treatment with PBS, PTX, EL, and ELP groups. (**e**) Body weight change of tumor-bearing mice. *** *p* < 0.001, **** *p* < 0.0001.

The in vitro investigations were performed to compare the antitumor efficacy between PTX and ELP. A subcutaneous CT26 tumor model was established to evaluate the therapeutic potency of ELP. CT26 tumor-bearing mice were randomly divided into four groups (*n* = 5). When tumor volume reached 100 mm^3^, the mice were intratumorally administered PTX, EL, or ELP at a concentration of 6 mg/kg PTX. A schematic representation of the treatment regimen was delineated in Figure 4a. Tumor specimens were excised and photographed on day 16 (Figure 4b), while tumor growth curves for each group were documented (Figure 4c). The results showed that both PTX and EL vectors could significantly reduce tumor growth rate compared to the PBS control group, but there was no significant difference between them. Compared with free PTX and EL vectors, ELP had a more significant antitumor effect.

**Figure 5:**
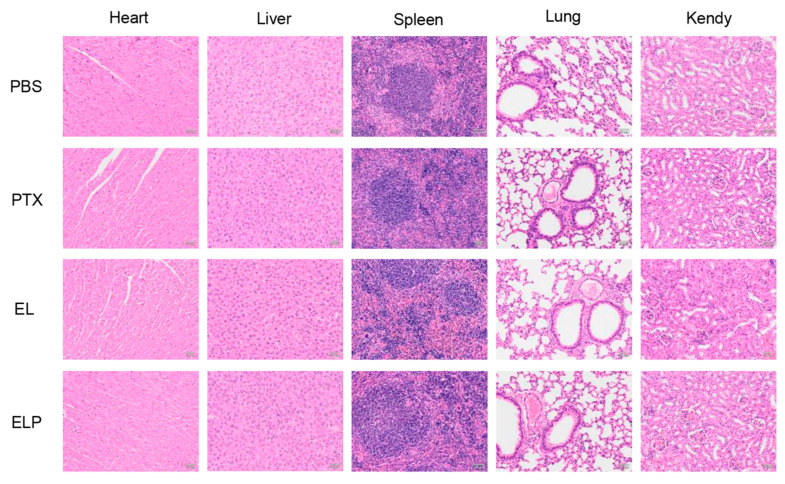
HE stains and immunofluorescence images of each group. Scale bar = 50 μm.

In the survival analysis, mice underwent identical treatment protocols, with endpoints defined as mice dying or tumor volume exceeding 3000 mm^3^ (Figure 4d). Notably, the administration of ELP significantly prolonged the survival of mice. Moreover, there were no appreciable discrepancies across all groups in the body weight of mice (Figure 4e). Histological evaluation of cardiac, pulmonary, splenic, and renal tissues revealed an absence of significant pathological alterations across all treatment cohorts (Figure 5). Thus, relative to free PTX and EL, ELP conferred enhanced antitumor effects without eliciting severe adverse events.

### 2.5. The Ability to Modulate Tumor Immune Microenvironment

**Figure 6:**
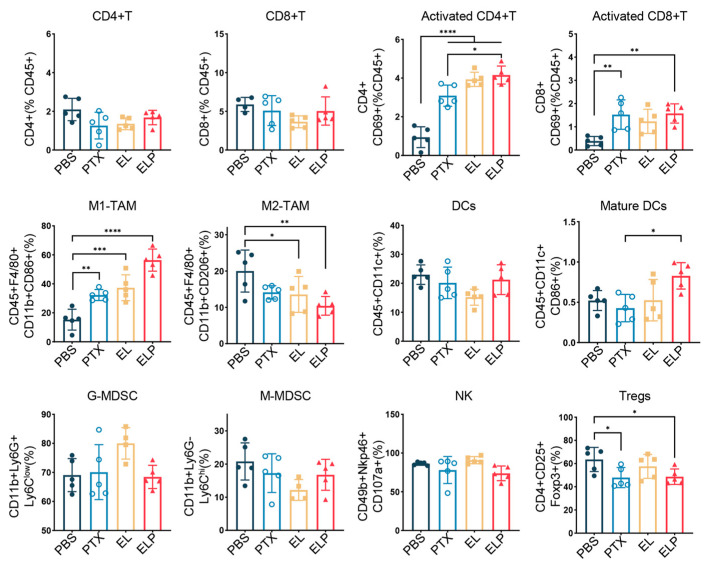
In vivo intratumoral immunity activation. Percentages of intratumoral lymphocytes, CD4^+^ T cells, CD8^+^ T cells, activated CD4^+^ T cells, CD8^+^ T cells, M1/M2 TAMs, DCs cells, mature DCs, G/M MDSC cells, NK cells, and Tregs in CT26 tumors were analyzed by flow cytometry (*n* = 5). * *p* < 0.05; ** *p* < 0.01; *** *p* < 0.001; **** *p* < 0.0001.

Tumor-associated immune cells are pivotal in orchestrating surveillance mechanisms focused on tumor recognition and eradication. To elucidate the underlying mechanisms driving the antitumor effects of EL and ELP, we used a CT26 tumor-bearing mice model to study the effects on the tumor immune microenvironment by intratumorally injecting PTX, EL, and ELP (Figure 6). Our findings revealed that both PTX and ELP treatment significantly augmented the population of activated CD4^+^ and CD8^+^ T cells within the CT26 model compared to PBS controls. Moreover, ELP administration notably enhanced the activation of CD4^+^ T cells in comparison to PTX treatment alone. Additionally, EL treatment also leads to increased activation of CD4^+^ T cells and, to a certain extent, CD8^+^ T cells relative to PBS controls.

Prior studies have reported that PTX can modulate tumor-associated macrophages (TAMs) towards the M1 phenotype studies [16]. Our results confirm these findings and further demonstrate that EL has a similar effect. Compared to PTX, ELP could further enhance polarization toward the M1 phenotype and reduce the M2 phenotype of TAMs. Furthermore, we assessed the status of dendritic cells (DCs), natural killer cells (NKs), myeloid-derived suppressor cells (MDSCs), and regulatory T cells (Tregs) within the immune microenvironment. Our results indicated that ELP treatment enhanced the activation of DCs compared to the PTX group and decreased the influence of Tregs. However, no significant differences were observed in the levels of MDSCs and NK cells. These findings collectively suggested that the observed antitumor effect of ELP was at least partly attributed to the activation of the intertumoral immune response.

## 3. Discussion

Due to their regenerative capabilities, mesenchymal stem cells (MSCs) and their derived exosomes were mostly used in wound healing and inflammation relief [17,18]. MSC-derived exosomes have shown potential in regulating tumors and serving as effective drug delivery vehicles for anticancer therapeutics. For instance, MSC-derived exosomes have been reported to decrease the mRNA levels of Aquaporin-5 and EGFR in colorectal cancer cells, both of which are important signaling molecules regulating tumor proliferation and metastasis [19]. Moreover, MSC-derived exosomes or mimetics have been demonstrated to act as carriers for anticancer drugs such as doxorubicin and paclitaxel (PTX), exhibiting inhibitory effects on tumor cell proliferation in vitro [20,21]. However, traditional exosomes encounter challenges in drug loading capacity and stability, which limit their effectiveness as drug carriers. In this study, we addressed these limitations by fusing MSC-derived exosomes with liposomes, resulting in a hybrid delivery system with improved properties as a carrier for anticancer drugs. Our findings reveal that hybrid exosomes significantly enhanced the stability and drug-loading capacity of exosomes. The hybrid exosome–liposome complexes also demonstrate superior performance as PTX carriers.

**Scheme 2:**
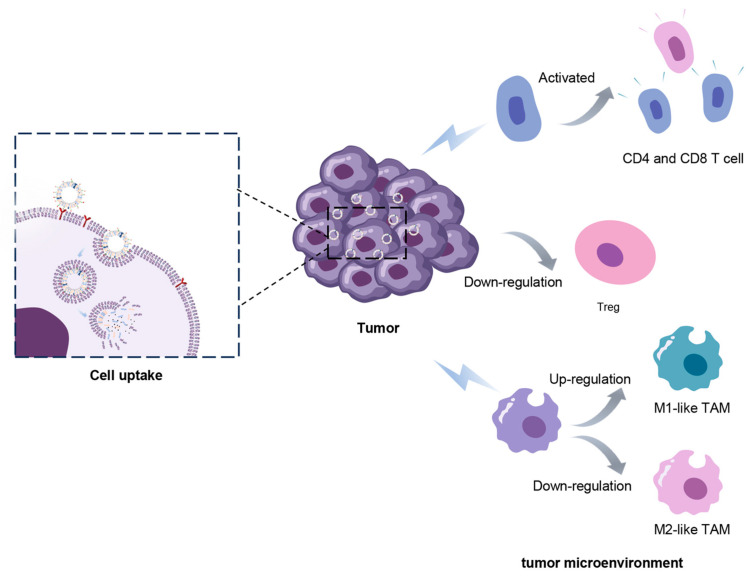
Illustration of the antitumor mechanism induced by ELP.

Although various anticancer therapies or combination treatments have been developed in current oncology practice, the prognosis of cancer patients remains poor. One of the primary reasons for the poor response of most tumors to chemotherapy drugs is the relatively low level of intratumoral immune activity during treatment, often referred to as “cold tumors” [22]. In the tumor microenvironment, the infiltration of various immune cells plays a pivotal role in tumor progression. While certain immune cell types such as neutrophils, macrophages, and CD4^+^ regulatory T cells have been observed to facilitate tumor growth, others like CD8^+^ cytotoxic T cells, natural killer cells, and gamma-delta T cells actively engage in tumor eradication. This intricate balance of immune cell dynamics underscores their clinical significance in colorectal cancer (CRC). The previous studies revealed a positive correlation between elevated levels of CD8^+^ T or M1 macrophage cells and improved overall survival rates in CRC patients [23,24]. Moreover, recent investigations into metastatic gastrointestinal esophageal adenocarcinoma (GEA) uncovered diminished levels of CD8^+^ T cells within metastatic lesions compared to primary tumor sites [25]. These findings highlight the critical role of immune cell infiltration in shaping the tumor microenvironment and influencing disease progression. Recent studies have highlighted that tumor-derived exosomes play a crucial role in promoting tumor development through various processes, including facilitating tumor cell epithelial–mesenchymal transition (EMT), inducing angiogenesis, mediating immune evasion, and regulating macrophage polarization [26]. Exosomes derived from immune-associated cells have the ability to enhance drug sensitivity and modulate the immune microenvironment. For instance, Lou et al. reported that intratumoral injection of ADMSC-derived miR-122-carrying exosomes significantly enhances the antitumor efficacy of sorafenib and inhibits tumor growth in hepatocellular carcinoma in vivo [27]. In our study, in vivo evaluation further substantiated the enhanced antitumor efficacy of ELP, with significant tumor growth inhibition observed in ELP-treated mice compared to those treated with free PTX or EL alone. Importantly, ELP treatment prolonged the survival of tumor-bearing mice without causing significant adverse effects, underscoring its safety and therapeutic potential. Mechanistic insights revealed that ELP treatment modulated the tumor immune microenvironment, leading to increased activation of CD4^+^ and CD8^+^ T cells, as well as polarization of tumor-associated macrophages towards the M1 phenotype. Additionally, ELP treatment augmented the activation of dendritic cells while reducing the influence of regulatory T cells, indicating its ability to stimulate an intratumoral immune response conducive to tumor eradication (Scheme 2). Our results demonstrated that the synthesized liposome–exosome drug delivery system not only inhibited the in vivo growth of CRC tumors but also activated the tumor’s immune microenvironment, reducing tumor immune evasion.

Thus, we have validated the advantages and feasibility of this platform for overcoming the limitations of exosomes in our research. By hybridizing exosomes with liposomes, we can enhance drug loading capacity and stability, as well as facilitate surface modifications on exosomes. However, there are also some limitations in our study. The selection of only the CT26-bearing mouse model for confirmation of CRC progression and immune response could have selection bias. Engineering exosomes for drug delivery still faces numerous challenges in clinical application. Our future research intends to construct an in-situ mouse tumor model to explore the function of the hybrid exosome drug delivery system in tumor progression and immune microenvironment regulation and aims to explore more efficient methods for exosome extraction and targeted modifications to enhance treatment efficacy.

## 4. Materials and Methods

### 4.1. Materials

#### 4.1.1. Cell Lines and Animals

The cell lines utilized in this study were purchased from the American Type Culture Collection (ATCC, Manassas, VA, USA), including the adipose-derived mesenchymal stem cell line MSC, human embryonic kidney 293T cells, mouse Colon Carcinoma cell line CT26, the mouse melanoma cell line B16, and human ovarian cancer cell line A2780. B16, CT26, and A2780 were cultured in Roswell Park Memorial Institute 1640 medium (Invitrogen, Carlsbad, CA, USA) supplemented with 10% heat-inactivated fetal bovine serum (FBS), 100 U/mL penicillin, and 100 mg/mL streptomycin. Notably, the human mesenchymal stem cell (MSC) line was nurtured in MSC NutriStem^®^ XF Medium (Biological Industries, Kibbutz Beit Haemek, Israel), complemented with 100 U/mL penicillin and 100 mg/mL streptomycin.

Female Balb/c mice aged 4 to 6 weeks, obtained from Liaoning Changsheng Biotechnology Co., Ltd. (Benxi, China), were utilized in the experimental procedures. The mice were housed under a 12 h light/dark cycle. Normal feed and water were supplied ad libitum. All animal handling protocols were adhered strictly to the Guidelines for the Care and Use of Laboratory Animals as delineated by the National Institutes of Health and were approved by the University Committee on the Use and Care of Animals of Jilin University.

#### 4.1.2. Reagents and Antibodies

The lipids 18: 1TAP (1, 2-dioleoyl-3-trimethylammonium-propane), DOPE (1, 2-dioleoyl-sn-glycero-3-phosphoethanolamine), EPC, 16:0 NBD PE, and 16:0 Liss Rhod PE were purchased from Avanti Polar Lipids, Inc. (Alabaster, AL, USA). DSPE-PEG2000-Folic acid was purchased from Nanocs (Boston, MA, USA). Chol (Cholesterol), Collagenase, and DNase I were purchased from Sigma-Aldrich (St. Louis, MO, USA).

The dye DiI was purchased from Beyotime Biotechnology. PTX was purchased from Meilunbio^®^ (Dalian, China). Paclitaxel injection was purchased from HAPHARM Group Co., Ltd. (Harbin, China). The CD9, CD63, and TSG101 antibodies were purchased from Abcam (Cambridge, MA, USA).

The antibodies of flow cytometry (anti-mouse): PE-CD45, FITC-CD4, APC-CD8a, PerCP/Cyanine5.5-CD69, FITC-CD3ε, APC-CD49b (pan-NK cells), APC/Cyanine7-CD335 (NKp46), PE-CD107a (LAMP-1), APC-CD45R/B220, PE/Cyanine7-CD69, FITC-CD45, APC/Cyanine7-F4/80, PE-CD11c, PE/Cyanine7-CD86, APC anti-mouse/human CD11b, PerCP/Cyanine5.5-Ly-6G, FITC-Ly-6C, PE/Cyanine7-CD45, APC-CD25, PE-FOXP3, PE-CD86, and PerCP/Cyanine5.5-CD206 (MMR) were purchased from Biolegend (San Diego, CA, USA).

### 4.2. Preparation and Characterization of the ELP Hybrid Exosomes

#### 4.2.1. Isolation of Exosomes

In this investigation, exosomes derived from mesenchymal stem cells (MSCs) were extracted from conditioned media through ultracentrifugation. Briefly, MSCs were cultured in exosome-depleted MSC NutriStem^®^ XF Medium, which had been centrifuged at 120,000× *g* before use. The supernatants were harvested and sequentially centrifuged at 300× *g* for 10 min, 2000× *g* for 20 min, and finally 10,000× *g* for 30 min to eliminate cells, cellular debris, and fragmented organelles. Exosomes were sedimented via ultracentrifugation at 120,000× *g* for 120 min at 4 °C. The resulting pellets were washed with phosphate-buffered saline (PBS), subjected to additional ultracentrifugation, and resuspended in sterile PBS. Protein concentrations of the exosome suspensions were quantified using the BCA assay and adjusted to 1 mg/mL. Isolated exosomes were preserved at −80 °C. Exosome markers CD63, CD9, and TSG101 were detected via Western blot analysis. The size distribution and zeta potential of MSC exosomes were determined using a Malvern Nano ZS90 instrument (Malvern Instruments, Malvern, UK).

#### 4.2.2. Preparation of the Folate-Modified Liposomes

The basic folate-modified liposomes were fabricated via the thin-film hydration method with a molar ratio of DOTAP/DOPE/CHOL/DSPE-PEG2000-Folic acid as 5/3/2/0.5. In the case of PTX-loaded liposomes, PTX was incorporated at a drug-to-lipid molar ratio of 1:30. Free PTX was removed through low-speed centrifugation at 3000 rpm for 10 min. For anionic liposomes, PC was substituted for DOTAP. DiI-labeled liposomes were introduced into liposomes at a concentration of 5 µM for cellular uptake experiment. Additionally, fluorescently labeled liposomes were formulated for FRET assays, wherein a 2% molar ratio of DOPE was replaced with 1 mol% 16:0 NBD PE serving as an electron donor and 1 mol% 16:0 Liss Rhod PE as an electron acceptor. All liposomal formulations underwent extrusion through polycarbonate membranes with pore sizes of 800 nm, 400 nm, and 200 nm. The size distribution and zeta potential of the liposomes were assessed using a Malvern Nano ZS instrument (Malvern Instruments).

#### 4.2.3. Preparation of the Hybrid Exosomes

The hybrid exosomes were generated through repeated freeze–thaw cycles performed 10 times with a mixture of exosomes and liposomes, with or without paclitaxel (PTX), and subsequently extruded through polycarbonate membranes with pore sizes of 400 nm, 200 nm, and 100 nm. For the preparation of DiI-labeled hybrid exosomes, the dyes were added at a concentration of 5 µM and then incubated at 37 °C for 20 min in the dark. Unentrapped dyes were removed by ultracentrifugation at 120,000× *g* for 120 min at 4 °C.

### 4.3. Western Blotting

MSC exosome lysates were clarified via centrifugation and subsequently separated by SDS-PAGE. The resulting gels were then semi-dry transferred onto nitrocellulose membranes (Bio-Rad Laboratories, Hercules, CA, USA). Following this, the membranes were subjected to blocking with 5% (*v*/*v*) nonfat milk in PBS at room temperature for 1 h. Subsequently, they were incubated with primary antibodies overnight, followed by three washes with PBST. Afterward, the membranes were exposed to secondary antibodies at room temperature for 2 h, followed by three additional washes with PBST. Protein bands were visualized using Tanon Highsig ECL Western blotting substrate within a chemiluminescent imaging system (Tanon 4600; Tanon, Shanghai, China) and analyzed with ImageJ 1.8.0 (NIH, Bethesda, MD, USA).

### 4.4. TEM

Five microliters of nanoparticles were individually subjected to negative staining using 1% phosphotungstic acid in dH2O (freshly prepared). Following staining, they were washed three times with dH2O, gently drained, and subsequently examined using transmission electron microscopy (TEM) at an accelerating voltage of 100,000 eV with a JEM-2100 instrument (Jeol, Tokyo, Japan)

### 4.5. Fusion Efficiency of the Hybrid Exosomes

The fusion efficiency of the Exo and Lip was verified by a fluorescence resonance energy transfer (FRET) study [28]. Samples were analyzed by fluorescence spectroscopy (LS55, PerkinElmer, Waltham, MA, USA) by exciting samples at 460 nm and measuring the emission spectra between 500 and 700 nm. Fluorescence resonance energy transfer (FRET) assays were employed to assess the fusion efficiency between Exo and Lip. According to the principle of FRET, NBD served as the donor molecule, while Rhodamine (Rho) acted as the acceptor molecule. DOPE in liposomes was replaced by NBD and Rho labeled PE lipids. Briefly, membrane fusion can increase the fluorescence intensity at 530 nm and decrease the intensity at 588 nm. After 10 freeze–thaw cycles, the samples were tested by exciting at 460 nm, and the emissions were measured at 588 nm and calculated by Equation (1). Liposomes and exosomes were fused at volume ratios of 1:1, 1:2, 1:4, and 1:6. After 10 freeze–thaw cycles, the samples were tested by exciting at 460 nm and measuring the emissions at 588 nm and calculated using the following:

Fusion efficiency (%) = (F_t_ − F_0_)/ (F_max_ − F_0_) × 100%, (1)

where F_t_ = emission fluorescence of test samples; F_max_ = emission fluorescence of max fusion by adding 0.2% volume of Triton; and F_0_ = emission fluorescence of alone liposomes.

### 4.6. Stability of Different Nanoparticles

The stability of Exo, Lip, and EL over four weeks was assessed using Malvern Nano ZS (Malvern Instruments). Briefly, the particle size and zeta potential of various nanoparticles were measured and recorded weekly post-preparation. For stability in fetal bovine serum (FBS), samples were introduced into a solution comprising 10% FBS and 90% 1640 medium at 37 °C with agitation at 100 rpm. Samples were collected at predetermined intervals (0, 0.5, 1, 2, and 4 h), and the particle size and Polydispersity Index (PDI) values of the corresponding samples were promptly characterized via dynamic light scattering (DLS).

For the hemolysis assay, blood was drawn into anticoagulant tubes to prevent clotting and centrifuged at 10,000× *g* for 10 min. The supernatant was discarded, and red blood cells (RBCs) were washed 3 times with PBS (pH 7.4). To evaluate the hemolytic effects, RBCs were incubated with varying concentrations of nanoparticles. The tubes were then incubated for 2 h at room temperature, followed by centrifugation for 5 min. Subsequently, 100 µL of supernatant was carefully transferred from each tube to a clean 96-well plate, and the absorbance of hemoglobin was measured at 540 nm. The positive control consisted of deionized water, while the negative control comprised PBS. The percentage of hemolysis was determined using the following equation [29]:

Percentage of hemolysis (x) = (optical density − negative control optical density)/(positive control optical density − negative control optical density) × 100.(2)

### 4.7. Drug Loading and In Vitro Drug Release

The concentrations of PTX were determined via high-performance liquid chromatography (HPLC) utilizing a UV/VIS detector set at 227 nm. To ascertain the drug loading content (LC) of different nanoparticles, 100 µL of nanoparticles was combined with 1 mL of acetonitrile (J.T. Baker) and subjected to ultrasonic bath treatment for 10 min to demulsify before analysis. Quantification of the components was performed using a C18 precolumn (Agilent, Eclipse XBD-C18, 4.6 × 150 mm, 5 µm), and the mobile phase comprised a mixture of acetonitrile and water (60: 40). The chromatographic method was validated in accordance with the requirements of the Chinese Pharmacopoeia, including specificity, limit of quantitation, limit of detection, intra- and inter-day precision, and others. All R^2^ values exceeded 0.9990 in the standard curves. The mobile phase composition consisted of 60% acetonitrile and 40% deionized water, with a flow rate of 0.8 mL/min.

The formulas for calculating LC (%) were as follows:

LC (%) = (Amount of PTX in vesicles/Total amount of vesicles) × 100%(3)

For drug release assays, PBS containing 1 mM sodium salicylate was utilized as the release medium, and 3.5 kDa dialysis tubes were employed at pH 7.4. The release medium was stirred at 150 rpm, and 1 mL of samples was withdrawn at specified time intervals (0.25, 0.5, 1, 2, 4, 8, 12, 18, 24, 30, 36, and 48 h). Volumes were replenished with fresh release medium after each sampling. The samples were filtered through a 0.22 μm syringe filter, and the concentration of PTX was determined by HPLC (Agilent) following appropriate dilution with acetonitrile followed by the mobile phase. In vitro release experiments were conducted in triplicate for all formulations, and results were reported as the cumulative amount of drug released at each time point.

### 4.8. Confocal Laser Scanning Microscopy (CLSM)

A2780 cells, B16 cells, and CT26 cells were seeded onto glass coverslips in 24-well plates with 2% (*v*/*v*) serum medium. Each well contained 10^5^ cells, and the cells were incubated for 24 h. Exo and EL were incubated with 5 μM DiI for 1 h at 37 °C and then centrifuged at 120,000× *g* for 1 h to remove free dye. DiI-labeled Exo or EL were added to the wells, with MSC exosomes at a concentration of 20 μg/mL, and the cells were further incubated for 4 h and cell nuclei were stained with DAPI. Finally, the coverslips were examined by CLSM.

### 4.9. Cell Viability Assay

Cell death was assessed via an MTT (3-(4,5-dimethylthiazol-2-yl)-2,5-diphenyltetrazolium bromide) assay. B16 cells, CT26 cells, and A2780 cells were seeded at a density of 1 × 10^4^ cells per well in 96-well plates and cultured for 24 h. Subsequently, the culture medium was replaced with fresh medium containing PTX, Exo, EL, and ELP, and the cells were incubated for 24 h. Following incubation, 20 μL of MTT solution (5 mg/mL) was added to each well and incubated for an additional 4 h. Afterward, the supernatant was discarded, and the absorbance was measured at 490 nm using a microplate reader.

### 4.10. In Vivo Treatment

All animal procedures were conducted in adherence to the Guidelines for the Welfare of Animals in Experimental Neoplasia. Female BALB/c mice aged 6–8 weeks were subcutaneously injected with 10^6^ CT26 cells and then randomly allocated to four groups, with a mean final tumor volume of 100 mm^3^. The mice received intratumoral injections of PBS, PTX, EL, or ELP every 3 days four times. Tumor diameters were measured every two days using a digital caliper, and tumor volume was calculated using the formula length × width^2^ × 0.5. Throughout the study, both tumor sizes and individual animal body weights were continually monitored. On day 16, five mice in each group were randomly collected for tumors and their organs were excised and fixed in 4% (*v*/*v*) formaldehyde. Specific tissue sections were stained with hematoxylin and eosin (H&E) and then observed and photographed under a fluorescence microscope.

### 4.11. Flow Cytometry

For in vitro apoptosis assays, cells were added with different nanoparticles at 1 μM PTX concentration, collected at 24 h, and washed with PBS. Cells were resuspended in binding buffer, stained with Annexin V for 15 min and propidium iodide (PI) for 5 min in the dark at room temperature, and then analyzed by flow cytometry with an Accuri C6 flow cytometer (BD, Franklin Lakes, NJ, USA).

For tumor immune microenvironment analysis, five mice from each group were randomly euthanized at day 16, and tumor tissues were collected and weighed. Subsequently, the tumor tissues were incubated in RPMI 1640 medium containing 1 mg/mL collagenase (#C5138; Sigma-Aldrich, St. Louis, MO, USA) and 200 U DNase I (#D5025; Sigma-Aldrich, St. Louis, MO, USA) at 37 °C for 1 h to obtain single cells. One million tumor cells were then incubated for 30 min on ice in a staining medium with relevant antibodies for surface expression analysis with antibodies from Biolegend (San Diego, CA, USA). For intracellular staining of Foxp3 and CD206, fixation concentrate and diluent (#00-5521-00; eBioscience, San Diego, CA, USA) along with permeabilization buffer (#00-8333-56; eBioscience, San Diego, CA, USA) were utilized. Samples were acquired using the Accuri C6 flow cytometer (BD, Franklin Lakes, NJ, USA) and analyzed.

For intracellular staining, cells were fixed and permeabilized with Cytofix/Cytoperm buffer (BD Biosciences, San Jose, CA, USA) and washed with a 1× perm/wash solution (BD Biosciences) before incubation with relevant primary antibodies for 30 min. After washing, the samples were analyzed using a FACS Calibur flow cytometer (BD Biosciences, San Jose, CA, USA) with the FlowJo software (Version 10.8.1).

### 4.12. Histological Analysis and Immunohistochemistry

The excised tissues were fixed in 4% paraformaldehyde, dehydrated with ethanol, embedded in paraffin, and sectioned. Subsequently, the sections were stained with hematoxylin and eosin (H&E). Images were captured using an Olympus VS200 microscope.

### 4.13. Statistical Analysis

All data were processed in GraphPad Prism version 8.3 for Windows (GraphPad Prism 8 Software, La Jolla, CA, USA). Significant differences in the data were analyzed by *t*-test or ANOVA. The results were expressed as mean ± S.E.M. and are considered significant at *p* < 0.05.

## 5. Conclusions

In summary, our research has developed a novel MSC-derived exosome–liposome hybrid delivery system, which significantly improves the carrier stability and drug loading capacity of PTX. This PTX-loaded nano drug delivery system demonstrates enhanced treatment efficacy in colorectal tumor-bearing mice and, furthermore, modulates the tumor immune microenvironment, including increased activation of CD4^+^ and CD8^+^ T cells, M1 macrophage polarization, and Treg cell downregulation. Our hybrid system provides valuable insights into exosome engineering and holds highly promising prospects for future application.

## Data Availability

The data that support the findings of current research are openly available in the article. Raw and derived data underlying the findings of current research are available from the corresponding author upon reasonable request.

## Supplementary Materials

The supporting information can be downloaded at: https://www.mdpi.com/article/10.3390/ijms25073645/s1.
