# Supplementary material for: Enhanced Therapeutic Potential of Hybrid Exosomes Loaded with Paclitaxel for Cancer Therapy
Source: Int J Mol Sci. 2024 Mar 25;25(7):3645. doi: 10.3390/ijms25073645 (PMC11012016; doi:10.3390/ijms25073645)
Supplement: Supplementary file 1 [file ijms-25-03645-s001.zip › ijms-2918847-supplementary.pdf]

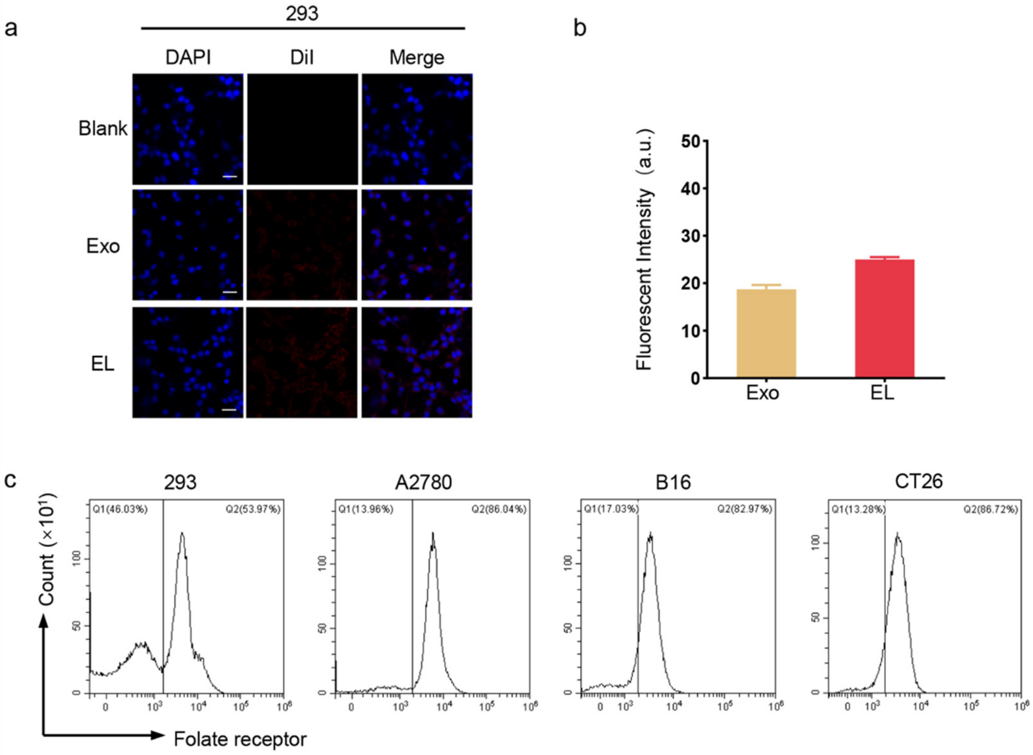

Figure S1. In vitro assays of different cell lines. (a) The CLSM of 293 cells. DiI, a lipophilic red fluorescence dye, was selected to label the Exo and EL. DAPI, a blue fluorescence dye, was selected to label the cell nucleus. Scale bar: 50 μm. (b) The quantification of CLSM by ImageJ software. (c) Flow cytometry of folate receptor expression of four different cells.
